# Impact of Six Years Community Directed Treatment with Ivermectin in the Control of Onchocerciasis, Western Ethiopia

**DOI:** 10.1371/journal.pone.0141029

**Published:** 2016-03-04

**Authors:** Abdi Samuel, Tariku Belay, Delenasaw Yehalaw, Mohammed Taha, Endalew Zemene, Ahmed Zeynudin

**Affiliations:** 1 Wollega University College of Medical and Health Sciences Program of Medical Laboratory Sciences, Nekemte, Ethiopia; 2 Jimma University College of Public Health and Medical Sciences, Department of Medical Laboratory Sciences, Jimma, Ethiopia; 3 Jimma University College of Natural Sciences, Department of Biology, Jimma, Ethiopia; 4 Jimma University College of Public Health, Department of Epidemiology, and Biostatistics, Jimma, Ethiopia; The University of Melbourne, AUSTRALIA

## Abstract

**Background:**

The African Program for Onchocerciais Control (APOC) with a main strategy of community directed treatment with ivermectin (CDTI) was established with the aim of eliminating Onchocerciasis as a disease of public health and socio-economic importance. The study area was a hyper endemic area just before the implementation of CDTI. It has been implemented for six years in this district but yet not been evaluated. So, the objective of this study was to evaluate the impact of six years CDTI on parasitological and clinical indices of Onchocerciasis

**Methods:**

This study employed a pre-post impact evaluation design. The minimum sample size for this study was 1318; the respondents were selected by multi-stage sampling technique. Data on socio-demographic characteristics using a semi-structured questionnaire, clinical examination for skin signs and symptoms of Onchocerciasis and two bloodless skin snips from each side of the gluteal fold were taken from the entire study participants. SPSS version 16.0 and Medcalc version 12.2.1.0 were used for analysis.

**Result:**

The microfilaridermia reduced from the pre-intervention value of 74.8% to 40.7%, indicating a 45.6% reduction, mean intensity from 32.1(SD = 61.5) mf/mg skin snip to 18.7(SD = 28.7)indicating 41.75% reduction, CMFL from 19.6 mf/mg skin snip to 4.7 indicating 76% reduction. The result also showed that microfilaridermia and mean intensity decreased as the number of treatment taken increased. Pruritis, leopard skin, onchocercomata and hanging groin reduced by 54.4%, 61.3%, 77.7% and 88.5% respectively.

**Conclusions:**

The implementation of CDTI significantly reduced the parasitological and clinical indices of Onchocerciasis, so, efforts should be made to improve the annual treatment coverage and sustainability of CDTI to drastically reduce the micro filarial load to the level the disease would no longer be a public health problem.

## Introduction

Human Onchocerciasis is caused by the filarial parasitic nematode *Onchocerca volvulus* which is spread by black flies belonging to the genus Simulium[1]. It has remained an important public health problem in Tropical Africa, Latin America, and Yemen. The African Onchocercal belt extending from Senegal in the West to Ethiopia in the East. The true extent of the disease has been estimated by Rapid Epidemiologic Mapping of Onchocerciasis (REMO) which estimated that 37 million people carry *O*. *volvulus*, with 90 million at risk in Africa [2].

In Ethiopia, Onchocerciasis has been known to be endemic in several localities of the Southwestern and Northwestern parts in different magnitudes of endemicity. A countrywide REMO carried out in 1997 and 2001 by APOC revealed that Onchocerciasis was much more widespread in Ethiopia than previously estimated [3]. In different studies conducted in Ethiopia the highest point prevalence recorded was 85.3% in Teppi coffee plantation [4, 5]. In a previous study conducted in four different villages of Anfilo District, Kelem Wollega Zone, Western Ethiopia or the current study area, the average prevalence of Onchocerciasis was 74.8%. Among the villages 87.6% was recorded in Yeli, which is the highest ever recorded in Ethiopia [6].

The microfilariae of *Onchocerca volvulus* are the main cause of the clinical manifestations of the disease. These include: dermal manifestations, irreversible ocular lesions resulting first in impaired vision and finally in total blindness, however ocular Onchocerciasis that causes loss of vision and blindness was considered to be mild in Ethiopia [7, 8]. In Ethiopia, the main symptom of the disease is dermal manifestations that are characterized by intense itching and thickening of the skin, hanging groin and depigmentation of the skin. Beyond the debilitating health burden, Onchocerciasis also inflicts tremendous social and economic damage on individuals and entire communities. It also reduces marriage prospects for both women and men [9].

Onchocerciasis is deemed responsible for the annual loss of approximately 1 million DALYs more than half of them due to skin disease, which greatly reduces income-generating capacity, incurs significant health expenditures, and exerts, overall, an immensely negative socioeconomic impact on the afflicted populations and their land use[2]. In line with this, in a case control study conducted in Ethiopia to determine the effect of non-blinding Onchocerciasis on work productivity at Baya farm; Teppi coffee plantation project, cases had significantly lower total house hold income and of average per capita income, cases were significantly more likely to be absent from their work for reasons of sick leave and unauthorized leave than controls, controls earned 25.2% increase over the salary earned by cases and spend a large portion of their income on extra health costs[10].

To overcome the burden of this disease African Program for Onchocerciasis Control (APOC), with a main strategy of community directed treatment with ivermectin, (CDTI) was established with the aim of eliminating Onchocerciasis as a disease of public health and socio-economic importance [11]. APOC’s strategy, CDTI, relies on active participation and promotes community ownership and empowerment of communities to distribute ivermectin treatment to people who need it. Communities take responsibility for ivermectin distribution and decide how, when and by whom the ivermectin treatment should be administered. This strategy has been implemented in the study area for six years but it was not evaluated, so the aim of this study was to evaluate the change in clinical and parasitological indices of Onchocerciasis based on pre and post treatment community based survey.

## Methods

### Study Settings and Period

Anfilo district is located 752km west of the capital Addis Ababa. It is found in the south western part of Kelem Wollega Zone at a distance of 42 km away from the Zonal Capital Dembi Dollo town. Mugi is the administrative capital of the district.

The major rivers draining the district include Ega, Chamo, Agami, Gati, Sabu, Yawi, Ursa and Yaga. The rivers are mostly used for irrigation (traditional/’bone’), drinking and sanitation purposes. Coffee is an important cash crop, Over 50 square kilometers are planted with this crop.

This study was conducted in four villages of Anfilo district where the base line study was conducted namely: Dola, Shebel, Waba, and Yeli from February to April, 2012. In this district CDTI have been implemented for six successive years. The annual therapeutic coverage for the communities over the six years period was ranged from 75% to 80%.

### Study Design

The study employed a pre-post impact evaluation design.

A pre intervention community based cross-sectional study to asses clinical and parasitological indices of Onchocerciasis was conducted just before the implementation of CDTI in 2006, for use in control program and for its subsequent evaluation [6]. During this time the study was conducted on a total of 1114 population and all the study participants were screened for dermatological manifestations of Onchocerciasis and two blood less skin snip were collected from the right and left gluteal fold for detection of the microfilaria by a trained health professionals.

For post intervention status community based cross-sectional study to assess clinical and parasitological indices of Onchocerciasis was conducted from February—April, 2012 just in the same method with the pre intervention study except that the total sample size in the post intervention case was 1318 and the other difference was that in the pre intervention study individuals of age ≥ 15 were included in the study but in the post intervention study individuals of age ≥ 3 years were included.

### Inclusion and Exclusion criteria

Consented and Assented individuals of age ≥ 3years and those who had lived in the area for at least one year were included in the study regardless of their treatment status.

### Sample size

Sample size was determined using the formula for determination of sample size for single population proportion as follows:

$$n={\left(Za/2\right)}^{2}\;\text{P}(1-\text{P})/{\text{d}}^{2}$$

n = minimum sample size, Z α/2 = level of significance

Confidence Level (CL) _= 95%_, Margin of error (d) = 4%, Prevalence of microfilaria (P) = 48%

48%

### Assumption made for calculating the minimum sample size

Prevalence (p) of Onchocerciasis in the study area at the base line was 74.8% but the intervention was implemented for six years in this area just after the result of this study. Therefore a reduction in prevalence was expected. So, a study conducted in Nigeria[12] was considered for the assumption because, one, the prevalence at the baseline was 74% which was comparable to the prevalence in the study area at the baseline, second, the study was conducted after six years of the intervention again which was similar to the current study and the therapeutic coverage was almost comparable with the current study area, therefore like this study a 35.9% reduction from the pre intervention prevalence was assumed Threfore, P for this study was 48% (0.48).

$$n={\left(1.96\right)}^{2}\;\times \;0.48(1-0.48)/{\left(0.04\right)}^{2}$$

n = 599

Since a multistage sampling technique was used the calculated sample size was multiplied by 2 for design effect and 10% non response rate was considered, so the final minimum sample size for the study was n = 599x2+120 = **1318**

### Sampling Techniques and procedures

This study used **a multistage sampling technique**. First four villages namely: Waba, Dola, Shebel and Yeli were selected purposively since there were a baseline study in these areas just before the implementation of the intervention and then the calculated total sample size (1318) was allocated proportionally to the population size of the villages: 622 for Shebel, 258, 133, and 305 for Waba, Yeli, and Dola respectively. After allocating the number of study participants for each village, the households included in the study in all the selected villages were selected by simple random sampling technique using a random number table. Finally study participants from each selected households was selected by lottery method.

### Data collection techniques and instruments

First, identification data on socio-demographic characteristics like, age, gender, marital status, occupation, number of years of resident and history of previous treatment for onchocerciasis were collected using a semi structured questionnaire translated to Afan Oromo, then each study participant underwent a clinical examination. The clinical examination for skin signs and symptoms of onchocerciasis such as palpable nodules on their bodies, pruritis, leopard skin and hanging groin was conducted by trained one health officers and one BSc nurses in a separate room with adequate illumination to maintain the privacy of study participants using Murdoch clinical classification and grading system of the cutaneous changes in onchocerciais (21).

Finally from each study participant’s two bloodless skin snips, one from each side of the gluteal fold was aseptically taken using disposable blood lancet and razor blade as per the standard operational procedure (SOP). Then skin snips were placed in eppendorf tubes containing 100 micro liter of physiological saline and incubated at room temperature for 24 h to assure complete emergence of microfilariae from the skin biopsies. After 24 h, 100 micro liter of 4% formaldehyde was added to preserve the morphological features of microfilaria (mf).

After blotting to remove excess moisture each skin biopsy was weighed using analytical balance then the fluid in each tube was thoroughly mixed and pipetted onto a glass slide for identification and counting of the mf under a microscope. The number of mf from each biopsy was expressed as mf per milligram (mf/mg) of skin snip. Positive samples were randomly selected and stained with Giemsa staining solution to differentially diagnose microfilariae of *O*. *volvulus* from *M*. *streptocerca*.

### Data analysis and processing

Finally data were checked for completeness manually, then coded and entered in SPSS version 16.0 and Medcalc version 12.2.1.0 for analysis. Frequencies and proportions were calculated for the descriptive data and the results were presented using tables and graphs. Differences in prevalence for pre and post treatment survey were tested using Chi-squared (X2) test. The comparison of intensity measures were tested by Student’s t-test.

The community microfilaria load (CMFL): the geometric mean number of microfilaria per skin snip among adults aged 20 years and over in the community, including those with negative counts. This mean was calculated using a log (x+1) transformation. Differences and associations were considered significant when P value less than 0.05 was obtained.

### Data Quality control

To assure the quality of the data the following measures were taken: training was given for supervisors and data collectors during the process of data collection, Pre testing of the questionnaire was under taken and all completed questionnaire were examined for completeness and consistency during data collection and analysis. Standard operating procedures were followed for the laboratory procedures. 5% of the tests were repeated randomly to check the accuracy and reliability of the working procedure and performance of the laboratory personnel to minimize technical and observer bias.

### Ethical consideration

The study was conducted after obtaining ethical clearance from Ethical review committee of Jimma University and after obtaining Authorization and official credential from the Zone, Woreda health bureau and from local community authorities. The objectives and procedures, risks and benefits of the study were explained to the participants or their guardians in Afan Oromo (language of the study community). Any necessary data for the study was collected from the survey participants after obtaining a written informed consent from the participant’s. In case of all minor participants a written informed consent (Assent) was given by a parent or their guardian on behalf of all minor participants. Fortunately it was time for the annual mass treatment schedule so the positive individuals were advised to take the treatment.

## Results

In this study two independent community based surveys were conducted, one prior to implementation of Community Directed Treatment with Ivermectin (CDTI) to generate epidemiological and parasitological data for use in control program of the disease and subsequent evaluation and the second, six years after the implementation to assess the impact of the control program.

During the baseline study a total of 1114 study participants of age ≥ 15 years were examined for microfilaria of *Onchocercus vulvulus* and onchocercal skin disease, where as in the later study a total of 1237 study participants of age ≥ 3years were included in the study to know the overall current status of Onchocerciasis in the study area. To assess the impact of the control program only 971 of the 1237 study participant’s age ≥ 15 years were used.

At the baseline, 74.8% of the 1114 study subjects examined were positive for the microfilaria of *O*. *volvulus* in their skin snips. After implementation of the intervention, 40.7% of the 971 study subjects examined were positive for the microfilaria in their skin snips, indicating a 45.6% reduction in the prevalence of infection, which was a significant difference statistically (X^2^ = 247.8, 95% CI 29.97, 38.1, p < 0.001).

At the baseline the highest prevalence of infection was in Yeli village (87.6%) and the lowest in Dola(62.6%). The prevalence of infection in Yeli after the intervention was 43.7%, representing 50% reduction (X^2^ = 87.86, 95% CI 43.05–53.15, p < 0.001) and it was 26.2% in Dola which indicated a 58.2% reduction (X^2^ = 84.02, 95% CI 28.84, 43.45, p < 0.001). In both cases the change in prevalence was statistically significant.

The prevalence of onchocerciasis in less than 15 years study participants were 10.5% but no infection observed in under five years study participants.

The overall mean intensity of infection at the baseline was 32.1(SD = 61.5), where as its value after the intervention was 18.7(SD = 28.7) representing a 41.75% reduction. The pre and post treatment difference in mean intensity of infection was statistically significant (t = 6.2, 95% CI 9.18–17.62, p < 0.001).

After the intervention, CMFL the most sensitive parasitological indicator of Onchocerciasis, was reduced from its pre intervention value of 19.6 to 4.7, indicating a 76% reduction (t = 83.64, 95% CI 14.55, 15.25, p < 0.001). (Table 1) There were also statistically significant reductions in CMFL all over the study villages. (Table 2)

**Table 1:** Change in Prevalence, AMI, and CMFL after the Implementation of 6 CDTI, Anfilo district, Kelem Wollega Zone, Western Ethiopia, February—April, 2010.

Parameters	Baseline	After CDTI	Reduction (%)	P—value	95% CI
**Microfilaridermia**	74.8	40.7	45.6	P < 0.001	(29.97,38.1)
**AMI**	32.1	18.7	41.8	P < 0.001	(9.18, 17.62)
**CMFL**	19.6	4.7	76	P < 0.001	(14.55,15.25)

AMI: arithmetic mean intensity, CMFL: Community Micro filarial Load.

**Table 2:** Change in CMFL among the study villages, Anfilo district, Kelem Wollega Zone, Western Ethiopia, February to April 2012.

Village	CMFL (2006)	CMFL (2012)	Reduction (%)
**Dola**	8.4	3.7	56
**Shebel**	33.4	4.6	80
**Waba**	16.7	5.4	67.7
**Yeli**	42.3	6.7	84.3
**total**	19.6	4.7	76

Out of the total 971 study subjects age ≥ 15 yrs, 877(90.3%) of them were treated at least once with ivermectin. The prevalence of infection decreased as the number of treatment taken increased. The prevalence was low (20.3%) in those treated six times where as high(59%) among subjects treated only once, this difference in the frequency of treatment was statistically significant (X^2^ for trend = 68.406, P < 0.001).

The arithmetic mean intensity of microfilaria decreased as the frequency of treatment increased The result of this study revealed that, the highest mean intensity (58.9) being in the untreated subjects and the minimum (3.94) in those treated six times.

The overall variance to mean ratio (VMR) at the base line was 117.8 and CV was 191.6, whereas after the intervention the VMR was 44 and CV was 153% which indicated a reduction in the value of the two dispersion parameters. Even if there was a reduction in values of these parameters, the value of VMR after the intervention was greater than one and that of CV greater than 100% which indicated over dispersed distribution of the organism.

Pruritis, the most common clinical feature of onchocerciasis was 64.3% in prevalence at the baseline and 24.5% after the intervention, implying a 62.3% reduction (X^2^ = 333.79, 95% CI 36.01–43.93, P < 0.001). Generally the prevalence of leopard skin, nodules and hanging groin significantly reduced after the intervention. (Table 3)

**Table 3:** Change in clinical indices of onchocerciasis in Anfilo District Kelem Wollega Zone February—April 2012.

Clinical Spectrum	Prevalence at baseline	Prevalence after CDTI	Change in percentage
Pruritis	64.3	29.3	54.4
Leopard skin	19.1	7.4	61.3
Onchocercomata	12.1	2.7	77.7
hanging groin	5.2	0.6	88.5

## Discussions

The present study indicated a significant decrease in parasitological and clinical indicators of onchocerciasis after six years of intervention but onchocerciasis still remains a disease of public health importance in West Ethiopia, Anfilo district. The endemicity level reduced from the pre intervention hyper endemic level to mesoendemic.

The overall prevalence of onchocerciasis in all studied villages significantly reduced from 74.8% of its pre intervention value to 40.5%, which is a 45.6% reduction. Not only the overall prevalence of infection that showed a significant reduction in the study but also a remarkable reduction in prevalence in all the villages studied but with varying degree of reduction. The differences among the villages could be due to differences in their therapeutic coverage and compliance rate. This decrement in prevalence of the microfilaria was in agreement with a study conducted in the lower cross river basin of Nigeria [12], which had nearly a comparable therapeutic coverage with the current study area, had annual treatment schedule and also conducted after six years of intervention. Similar effects were also observed in Yanomami communities in the Brazilian Amazon [13], and other studies [14], [15] and [16]. But the study in Yanomami communities in the Brazilian Amazon [14], had a biannual treatment schedule unlike the present study area.

The result of the current study also indicated an inverse relationship between frequencies of treatment and prevalence of infection. In addition, during the base line study, even if CDTI was not in place some of the study subjects were accessing Ivermectin from private pharmacies and the result showed microfilaria rate was significantly higher among non-treated and decreased in prevalence as the number of treatment taken increased [6]. Similarly the study conducted in Burundi [16] also indicated that most of the persons who were found negative in the survey conducted after the implementation of the intervention had taken Ivermectin 3 or 4 times.

A 41.75% reduction in the mean intensity of infection was observed in the current study area.

This means from the pre intervention value of 32.1 mf/mg of skin snip to 18.7 mf/mg of skin snip. Similarly a reduction in the mean intensity was also observed in a study conducted in Yanomami communities in the Brazilian Amazon[13] but the percentage of reduction was very much higher than the current study, which may be due to the fact that participants in this study were followed throughout the study period, so that they received all the six rounds of treatment which may bring a remarkable reduction than the current study and its biannual treatment schedule may also contribute to the difference. Similar effect was also seen in [15–17].

The present study also showed a reduction in intensity of infection with the number of treatment taken. This finding was also in agreement with a study conducted in Burundi [16].

Regarding CMFL, the most sensitive parasitological indicator of onchocerciasis, the study showed a significant reduction from its pre intervention value of 19.6 to 4.7, indicating a 76% reduction. In addition to a significant reduction in overall CMFL, there were also significant reductions in CMFL in all the studied villages. Similar finding was also observed in [12–14], [16].

After the intervention the VMR was 44, which was greater than one and the CV was 153% which was greater than 100%. The value of these indices indicated an over dispersed distribution of the organism within the human host. This result was in agreement with the study conducted in Nigeria [14]. This finding indicated that even if there is a significant reduction in different parasitological indicators of onchocerciasis in the study area there are some groups or area that remains untreated and could be serve as a potential source of transmission. Therefore these groups may serve as a potential source of transmission and may prolong the time of control by another additional fifteen years

In the current study, pruritis, the most common feature of onchocerciasis was 64.3% in prevalence at the base line and 24.25% after the intervention, implying a 62.3% reduction Onchocercomata (nodule) rate reduced by 81.9%, which is similar finding with [14], [18]. Other clinical spectrum of onchocerciasis like: leopard skin reduced by 68.3%, and hanging groin by 90%. This finding is unlike other studies [14], [18].

In conclusion additional efforts should be made to improve the annual treatment coverage, so as to drastically reduce the micro filarial load to the level the disease would no longer be a public health problem. Further studies on Compliance rate, sustainability of CDTI, strain of the parasite, entomological study and longitudinal data should be gathered annually to have a strong conclusion about the impact of the program since the current study used a pre—post impact evaluation design due to lack of longitudinal data elsewhere in the country.

## Supporting Information

S1 Text STROBE Checklist. (DOC)
